# Supplementary material for: Sustained-input switches for transcription factors and microRNAs are central building blocks of eukaryotic gene circuits
Source: Genome Biol. 2013 Aug 23;14(8):R85. doi: 10.1186/gb-2013-14-8-r85 (PMC4054853; doi:10.1186/gb-2013-14-8-r85)
Supplement: Additional file 5 — HTML Browsable Motif Output. Zipped folder containing all WaRSwap and FANMOD motif output, viewable in a web browser. [file gb-2013-14-8-r85-S5.ZIP › HTML_browsable_motif_output/WaRSwap_all_tair9/sigs_WSWRSLDraw-2000_all.pvals.heatmaps.html/motif_id_174_011101101_tftype_all_upstream_-1000_0.html]

```
BG_MODEL = Rnew2
MOTIF_ID = 174_011101101
TF_TYPE = all
UPSTREAM = -1000_0


PVals
FN_0.2	FN_0.4	FN_0.6	FN_0.8
dg_60.genes	0.248	0.003	0.261	0.013
dg_70.genes	0.191	0.002	0.237	0.02
dg_80.genes	0.534	0.013	0.328	0.011

ZScores
FN_0.2	FN_0.4	FN_0.6	FN_0.8
dg_60.genes	0.657	4.321	-0.05	-0.106
dg_70.genes	0.876	4.633	0.011	-0.143
dg_80.genes	-0.193	3.175	-0.488	-0.096

StDevs
FN_0.2	FN_0.4	FN_0.6	FN_0.8
dg_60.genes	204.679	20.17	1.715	0.151
dg_70.genes	197.091	19.33	1.561	0.14
dg_80.genes	163.41	16.358	1.427	0.12
```
